# Microtubule stabilizer reveals requirement of Ca^2+^-dependent conformational changes of microtubules for rapid coiling of haptonema in haptophyte algae

**DOI:** 10.1242/bio.036590

**Published:** 2019-01-30

**Authors:** Mami Nomura, Kohei Atsuji, Keiko Hirose, Kogiku Shiba, Ryuji Yanase, Takeshi Nakayama, Ken-ichiro Ishida, Kazuo Inaba

**Affiliations:** 1 Shimoda Marine Research Center, University of Tsukuba, 5-10-1 Shimoda, Shizuoka 415-0025, Japan; 2 Biomedical Research Institute, National Institute of Advanced Industrial Science and Technology (AIST), 1-1-1 Higashi, Tsukuba, Ibaraki 305-8565, Japan; 3 Graduate School of Life and Environmental Sciences, University of Tsukuba, 1-1-1 Tennoudai, Tsukuba, Ibaraki 305-8577, Japan

**Keywords:** Haptophyte, *Chrysochromulina*, Haptonema, Microtubule, Paclitaxel, Curvature

## Abstract

A haptonema is an elongated microtubule-based motile organelle uniquely present in haptophytes. The most notable and rapid movement of a haptonema is ‘coiling’, which occurs within a few milliseconds following mechanical stimulation in an unknown motor-independent mechanism. Here, we analyzed the coiling process in detail by high-speed filming and showed that haptonema coiling was initiated by left-handed twisting of the haptonema, followed by writhing to form a helix from the distal tip. On recovery from a mechanical stimulus, the helix slowly uncoiled from the proximal region. Electron microscopy showed that the seven microtubules in a haptonema were arranged mostly in parallel but that one of the microtubules often wound around the others in the extended state. A microtubule stabilizer, paclitaxel, inhibited coiling and induced right-handed twisting of the haptonema in the absence of Ca^2+^, suggesting changes in the mechanical properties of microtubules. Addition of Ca^2+^ resulted in the conversion of haptonematal twist into the planar bends near the proximal region. These results indicate that switching microtubule conformation, possibly with the aid of Ca^2+^-binding microtubule-associated proteins is responsible for rapid haptonematal coiling.

## Abstract (precis)

**Summary:** Microscopy observations and pharmacological experiments revealed that the rapid coiling of a non-motor microtubule-based motile organelle, the haptonema, is explained by conformational changes of microtubules, including twisting and writhing.

## INTRODUCTION

Haptophytes are the group of microalgae that are widely distributed in oceans. They show similarities to heterokonts in chloroplast structure and chlorophyll species but are classified into an independent phylum owing to several cytological properties, including the lack of mastigonemes on flagella and the presence of extracellular scales or coccoliths (Christensen, 1962; Andersen, 2004). A haptonema is a filiform organelle uniquely present in haptophytes (Parke et al., 1955). It extends from a position between the bases of two flagella, reaching up to more than 100 μm in some species (Gregson et al., 1993a,b). A variety of functions have been demonstrated for a haptonema, including attachment and gliding on a substrate, formation of food aggregates, food capture and transport, and reception of mechanical stimuli (Manton, 1967; Leadbeater and Manton, 1969; Kawachi et al., 1991; Kawachi and Inouye, 1995). When haptophytes receive mechanical stimuli, they fully coil the haptonema within only a few milliseconds. By contrast, ‘uncoiling’, the process to resume the extended state, is much slower than coiling. The coiled state is thought to be a low-energy form, because the haptonema is always coiled when it is detached or when haptophytes are dead (Estep and MacIntyre, 1989).

Haptonema coiling is inhibited by EGTA depletion of extracellular Ca^2+^ or by the addition of an inhibitor of Ca^2+^-induced Ca^2+^ release, indicating that it is triggered by Ca^2+^ influx followed by efflux from a Ca^2+^ store (Kawachi and Inouye, 1994). Upon receipt of a mechanical stimulus, haptonematal coiling accompanies a change in waveform and an increase in beat frequency of flagella, possibly from changes in intracellular Ca^2+^ concentrations. This results in a quick response to avoid the stimulus (Gregson et al., 1993b; Kawachi and Inouye, 1994). The mechanism for the cytoskeletal response to Ca^2+^ is not well understood, except that centrin is localized as a small dot-like structure at the distal tip of haptonematal microtubules (Lechtreck, 2004).

Ultrastructural observations so far show that six to seven microtubules pass in parallel through a haptonema. In cross section, they are arranged in a circle of ∼100 nm in diameter in the middle region of the haptonema, and in an arc-shape with invagination of endoplasmic reticulum at the basal region. The number of microtubules reduces to three at the distal most region (Manton, 1964, 1967, 1968; Gregson et al., 1993b). It is well known that microtubules of flagellar axonemes have post-translational modifications (Wloga and Gaertig, 2010), but the patterns of modifications of haptonema microtubules are different from those of axonemes (Lechtreck, 2004). The microtubules are surrounded by fenestrated cisternae in the major part of a haptonema beneath the plasma membrane. From thin-section electron microscopy observations, the circular arrangement of microtubules often changes to a crescent arrangement after coiling. An electron dense structure in the center of the microtubule ring and a structure that links neighboring microtubules are observed (Gregson et al., 1993b). However, no structure that potentially represents motor proteins, such as dyneins or kinesins, has been observed. Thus, the molecular mechanism for rapid coiling of haptonemata is completely unknown.

Here we examined the structure of haptonemata and the process of their coiling using a newly identified marine species of the genus *Chrysochromulina*. Although the structures of this haptonema share common properties to those reported for other species (Gregson et al., 1993b), we obtained new information regarding microtubule configurations in this haptonema. Furthermore, we found that a microtubule stabilizer, paclitaxel (taxol), inhibits haptonematal coiling, suggesting that structural changes of the microtubules are responsible for the induction of rapid haptonema coiling.

## RESULTS

### Morphological characterization of *Chrysochromulina* sp. NIES-4122

The genus *Chrysochromulina* (Prymnesiophyceae) is characterized by the development of a relatively long haptonema in the subclass Chrysochromulinaceae (Edvardsen et al., 2011). Here, we used a *Chrysochromulina* species that was collected in Tokyo bay in 2013. The cell strain was established by clonal culture. This species has no calcareous coccoliths but has organic scales with no spine (Fig. S1A–C) and is morphologically classified into the genus *Chrysochromulina*. However, the shapes of the scales are distinct from any known *Chrysochromulina* species. This species possesses a haptonema of up to ∼150 µm in length, which is a little longer than that in *Chrysochromulina* *simplex*, *Chrysochromulina* *acantha* and *Chrysochromulina* *hirta* (Fig. S1B; Kawachi and Inouye, 1995; *C. hirta* is changed to *Haptolina hirta* after Edvardsen et al., 2011). When compared with other *Chrysochromulina* species, such as NIES-1333, the haptonema of this species was more resistant to mechanical stimuli that cause detachment from the cell body. Coiling was partially inhibited by depletion of Ca^2+^ in artificial sea water and completely inhibited by chelating intracellular Ca^2+^ (Fig. S1D). The strain used in this study has been deposited with the National Institute for Environmental Studies (NIES), Japan, as *Chrysochromulina* sp. NIES-4122.

### Observation of haptonematal coiling by high-speed recording

The haptonema of *Chrysochromulina* sp. NIES-4122 occasionally showed coiling during observation under a light microscope. Gentle tapping of the microscope stage induced almost 100% haptonematal coiling (Movie 1). The coiling occurred very rapidly and was complete within 5–10 ms (Movie 2), which is considerably faster than that observed in *C. acantha* (10–20 ms; Leadbeater and Manton, 1971). In contrast, uncoiling was much slower, taking ∼480 ms to complete extension (Movie 3).

**Fig. 1.**
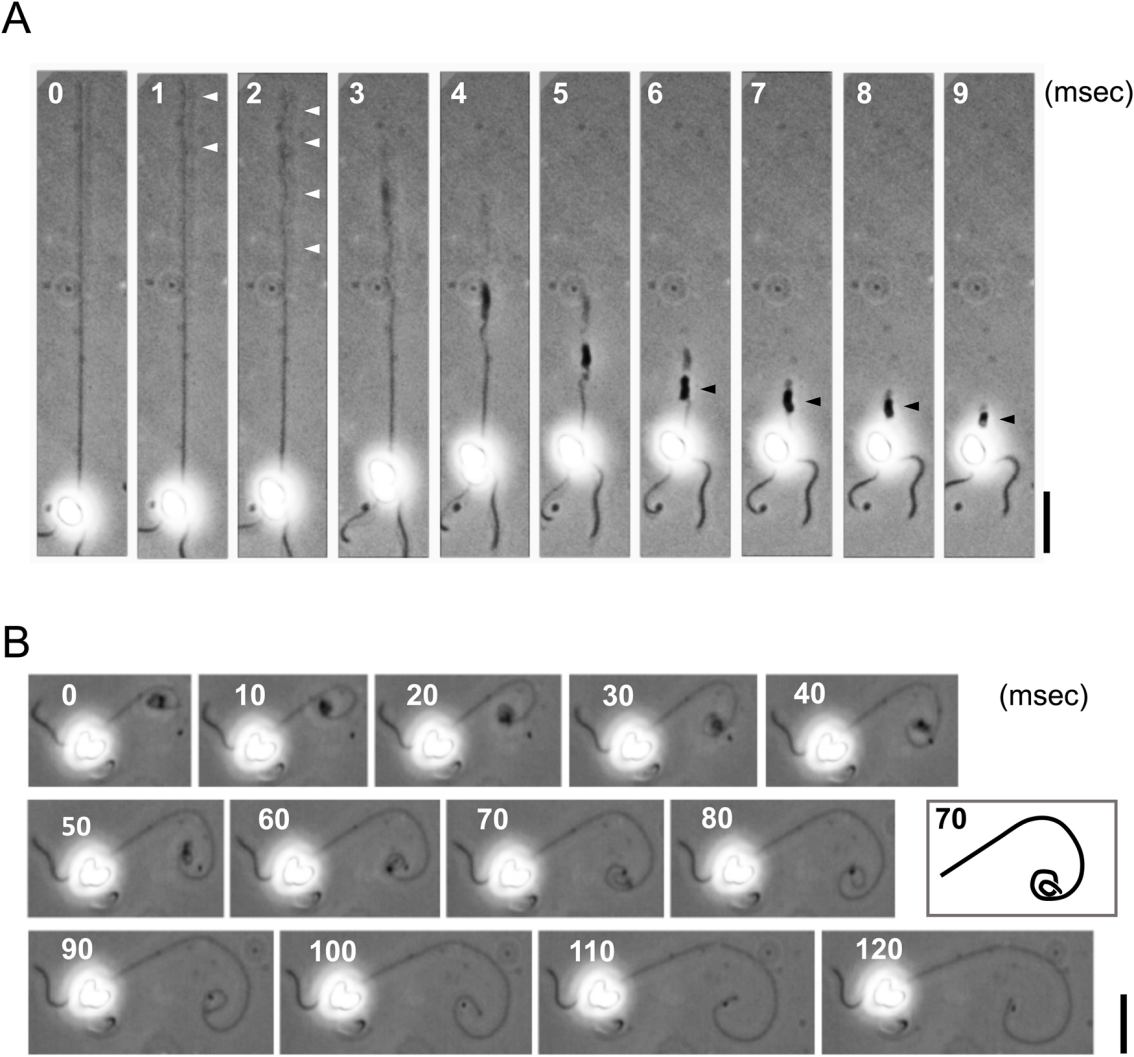
**High-speed analysis of haptonematal coiling and uncoiling.** (A) Coiling process of a haptonema. High speed images recorded at 1000 fps. Gentle helices that initially formed in the distal half of a haptonema are indicated by white arrowheads. Stacked coils are indicated by black arrowheads. Scale bar: 20 µm. (B) Uncoiling process of a haptonema. High speed images recorded at 200 fps. Scale bar: 20 µm. The inset represents the trace of the haptonema at 70 ms for clarification of the coiling direction.

One might expect that the coiling would start from the tip of a haptonema. However, detailed observation of the high-speed images revealed that this is not the case; the distal half of the haptonema first began to bend in gentle helices, followed by sequential coiling from the tip (Fig. 1A). The coil appeared to be left-handed, which was more clearly observed in the process of uncoiling (Fig. 1B). Uncoiling initiated from the proximal region of a haptonema while the distal most part remained curled, which was then gradually unwound during the last step of extension.

### Microtubule configuration in haptonemata in extended and coiled states

**Fig. 2.**
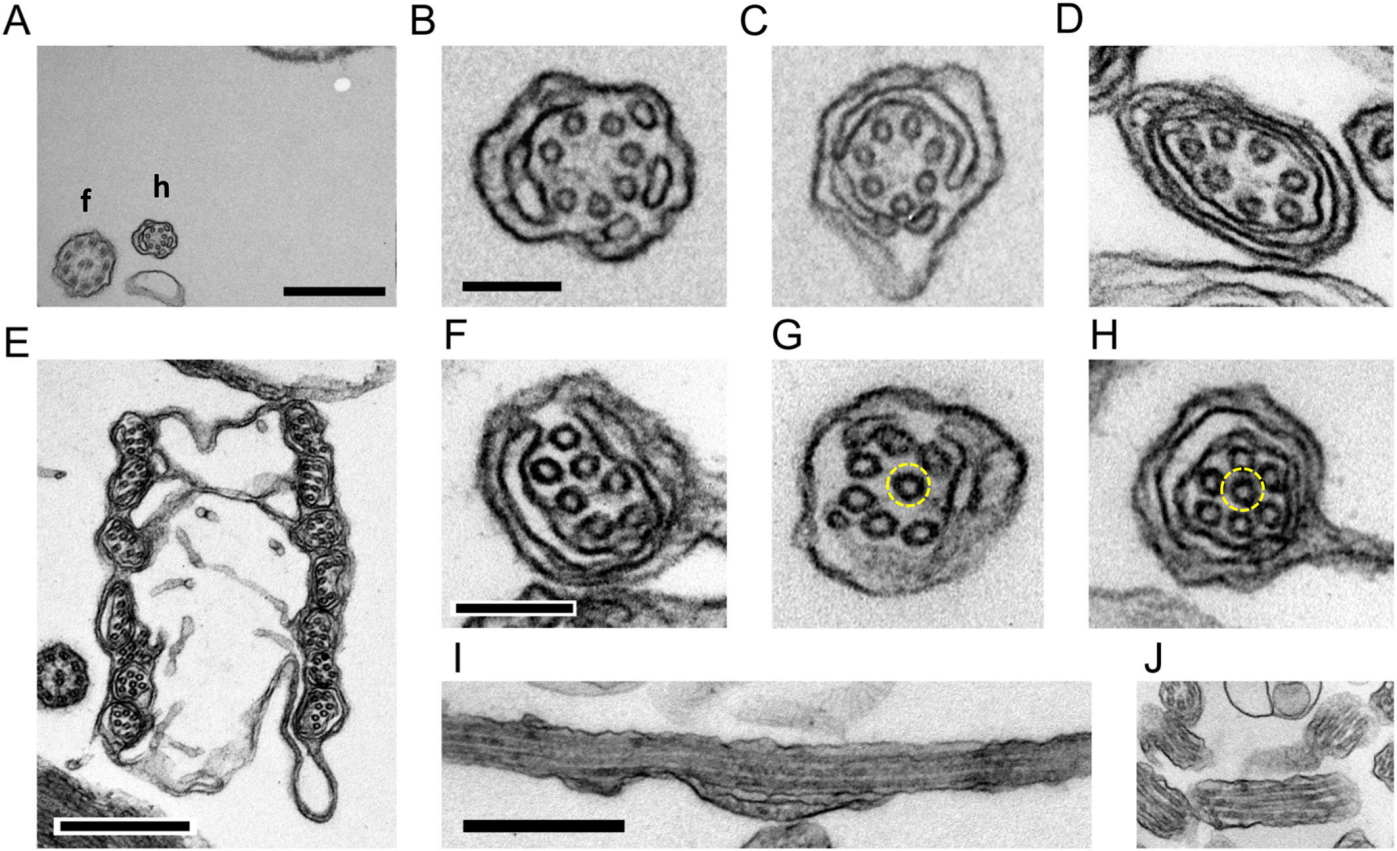
**Thin-section images of extended and coiled haptonemata.** (A) Extended haptonema at low magnification. Scale bar: 500 nm. (B–D) High magnification images of an extended haptonema. Scale bar: 100 nm. (E) Coiled haptonema at low magnification. Scale bar: 500 nm. (F–H) High magnification images of a coiled haptonema. Dashed yellow circles show a microtubule invaginated into the center. Scale bar: 100 nm. (I,J) Longitudinal images of extended (I) and coiled (J) haptonemata. Scale bar: 500 nm.

As reported in other species, including *Chrysochromulina* *chiton* (Manton, 1967), *C. simplex* and *C. acantha* (Gregson et al., 1993b), thin-section electron microscopy showed that in the extended haptonema seven microtubules are arranged in a ring, which is peripherally surrounded by cisternae (Fig. 2A–D). This circular arrangement of microtubules was distorted in the coiled haptonema and one of the microtubules was often invaginated towards the center (Fig. 2E–H). We measured the center-to-center distances between adjacent microtubules (Fig. S2A,B). The distances were relatively constant in an extended haptonema among sequential sections (Fig. S2C) but in a coiled haptonema one of the inter-filament distances sometimes became deviated, as if the ring was torn open (Fig. S2D). The deviated microtubule often changed its position relative to the adjacent microtubule (Fig. S2D). This pattern with an interfilament deviation distance of more than 15 nm was observed in 0% and 13% of extended and coiled haptonemata, respectively.

**Fig. 3.**
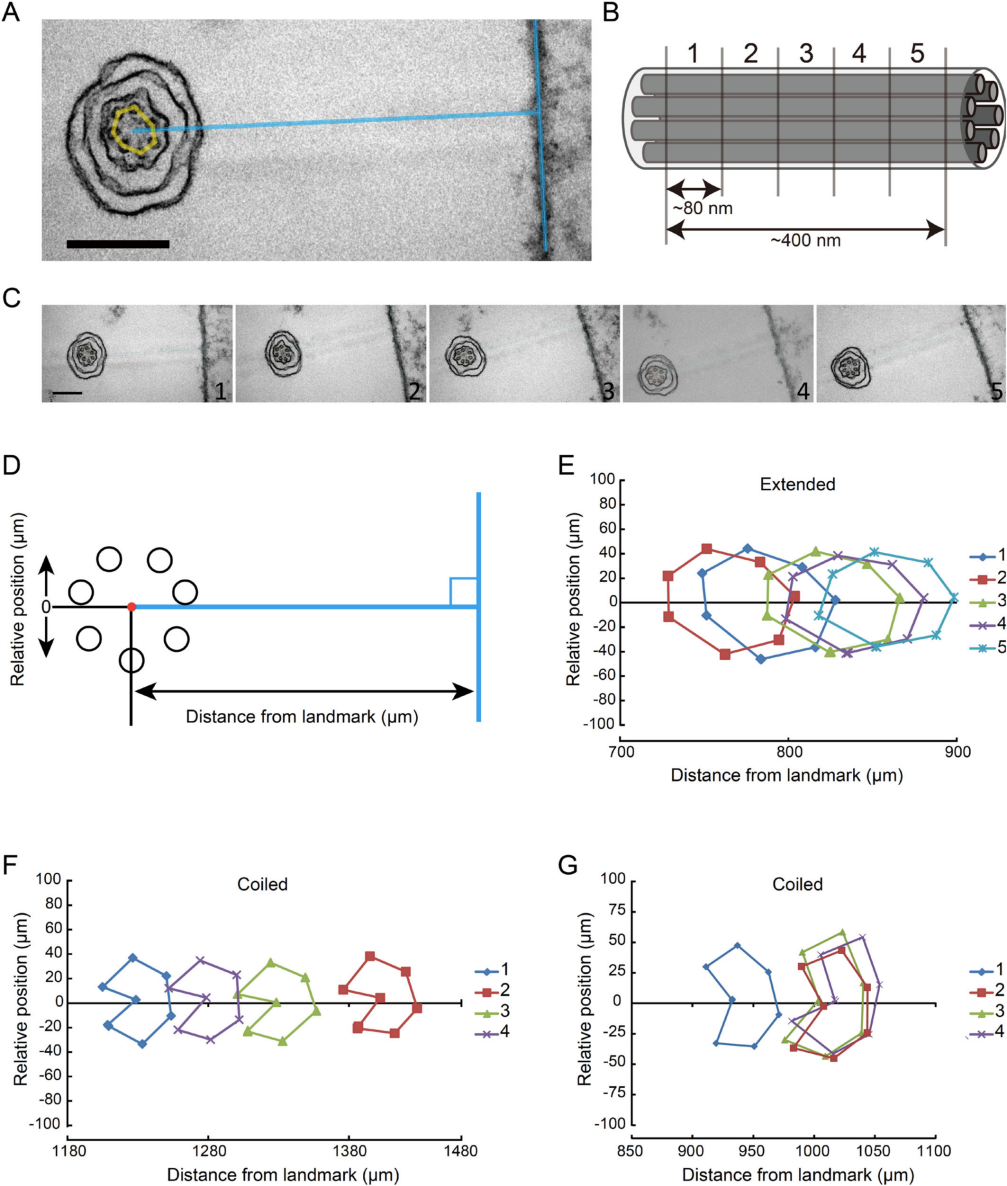
**Arrangement of microtubules along a haptonema.** (A) Extended haptonema adjacent to the poly-lysine-BSA coated Epon surface (landmark). To position each microtubule, a line was drawn from the center of the microtubule bundle at right angles to the surface. Scale bar: 200 nm. (B) Approximately 80 nm sequential sections were made. For example, five sections covered a 400 nm length of a haptonema along the longitudinal axis. (C) Example of sequential images of an extended haptonema. Scale bar: 200 nm. (D) Definition of the distance from the landmark and the relative position of each microtubule. (E) Typical plot of microtubule positions in five sequential images of an extended haptonema. (F) Typical plot of microtubule positions in five sequential images of a coiled haptonema. (G) Plot showing a helical arrangement of the seven microtubules in a coiled haptonema. Four out of 24 sets of sequential images of the coiled haptonema showed this pattern.

In longitudinal sections 70–80 nm thick, we were able to observe a parallel arrangement of three, sometimes four microtubules of up to 1 μm and 200 nm in extended and coiled haptonemata, respectively (Fig. 2I,J). This indicates that microtubules are arranged in a more or less parallel manner in both extended and coiled stages. To confirm the parallel arrangement of microtubules, we treated a small plate of polymerized Epon resin with poly-lysine. This was then coated with bovine serum albumin. Fixed haptophytes were deposited on the coated resin, post-fixed and embedded in Epon. This procedure provided a fixed landmark to measure the relative positions of each microtubule between sequential sections (Fig. 3A–C). The position of each microtubule relative to a landmark would change periodically if the haptonematal microtubules were arranged helically. In the case of parallel arrangement, the positions of seven microtubules would shift in parallel in the sequential sections (Fig. 3D). These results indicated that the microtubules are arranged in parallel, at least up to lengths of 400 nm and 320 nm for the extended and most of the coiled haptonemata, respectively (Fig. 3E,F). However, four out of 24 sequential images of the coiled haptonema showed twisted arrangements (Fig. 3G).

**Fig. 4.**
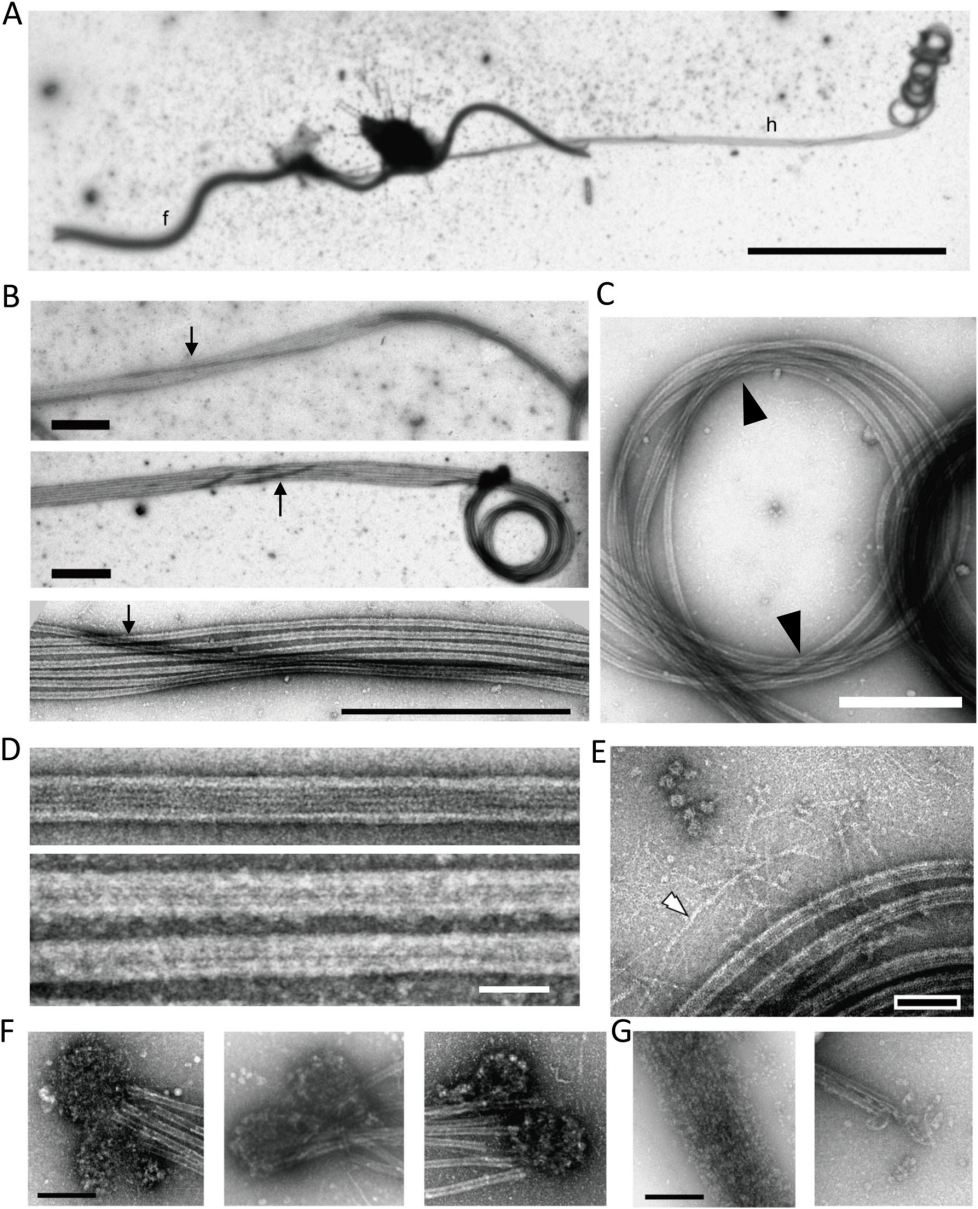
**Negative stain images of extended and coiled haptonemata.** (A) Negative stain image of a haptophyte. Because of demembranation, most parts of the cell body are removed. f, flagellum; h, haptonema. A long haptonema is observed on the right side with the tip coiled. Scale bar: 10 µm. (B) Three images showing that one, occasionally two microtubules (arrows), wind around the other microtubules in the extended region. Scale bar: 1 µm. (C) Coiled region. Microtubules are mostly parallel to each other, but there seems to be a twist with microtubules crossed over at the two opposite positions of the coil (arrowhead). Scale bar: 500 nm. (D) Microtubules polymerized from purified brain tubulin (upper panel) and microtubules in a demembranated haptonema (lower panel). Scale bar: 50 nm. (E) Image showing filamentous structures (white arrowhead) emanating from the microtubule bundle. Bar, 100 nm. (F) Mass of small particles observed at the distal tip of a haptonema. They are frequently observed in a pair. Scale bar: 200 nm. (G) Depolymerization of microtubules occasionally observed in demembranated haptonemata. The depolymerized microtubules are still bundled probably because of MAPs. The morphology of the small particles in F is similar to depolymerized tubulin dimers. Scale bar: 200 nm.

Next, haptophytes were deposited on a grid, demembranated by NP-40 and observed by negative staining electron microscopy (Fig. 4). The diameters of flagellar axonemes and haptonemata were distinct, so that a long haptonema could be distinctly observed at lower magnifications by negative staining (Fig. 4A). As reported by Gregson et al. (1993b), haptonematal microtubules were bound together after demembranation but were partly dissociated in some regions of the extended haptonema. Microtubules of the extended and coiled parts of a haptonema were mostly parallel to each other without any torsion. However, we often observed a microtubule loosely wound around the other six microtubules (Fig. 4B). This peculiar microtubule winding was not clearly observed in the coiled region where all the microtubules were mostly arranged in parallel. However, the microtubules in the coiled haptonema appeared not to be configurated as a simple coil. Instead, we observed the microtubules writhing and crossing over at two opposite positions in the coil (Fig. 4C, arrowheads; also see Fig. S7). This is compatible with the observations from thin-sectioned sequential images of some microtubule bundles being twisted in the coiled region (Fig. 3).

The diameter of the haptonema coil estimated from negative stain fixed images was 1.2±0.03 μm (*N*=83), similar to the value from differential interference contrast images of intact haptonema (0.96±0.03 μm, *N*=12). Accordingly, the microtubules in the coils had largely curved structures (Fig. 4C). To evaluate the mechanical property of haptonematal microtubules, we calculated the curvature of the image of the coiled haptonema by three-point method (Odde et al., 1999; Bicek et al., 2007; see Materials and Methods). The curvature of haptonematal microtubules in the coiled region was calculated as 1.76±0.55 rad/µm (*N*=35) using the negative stain images of microtubules. Similar values were obtained using coiled intact haptonema from differential interference contrast images (1.85±0.12 rad/µm, *N*=12). These results indicate that haptonematal microtubules have a high degree of curvatures that have not been observed with other microtubule structures, slightly beyond the value for microtubules at breakage (1.7 rad/µm in Waterman-Storer and Salmon, 1997; 1.5±1.0 rad/µm in Odde et al., 1999).

It is likely that binding of haptonema-specific microtubule-binding proteins (MAPs) prevents the curved microtubules from depolymerization. In fact, negative stain images comparing haptonematal microtubules (Fig. 4D, lower panel) and microtubules polymerized from purified tubulin (Fig. 4D, upper panel) showed that the haptonematal microtubules are covered with additional proteins. Sometimes, when a part of the microtubules seemed to have been depolymerized in a demembranated haptonema, a bundle of filamentous structures remained in place of microtubules (Fig. 4G, left panel). Since depolymerized tubulin molecules usually disperse in solution in the absence of MAPs, the structures observed after depolymerization of haptonematal microtubules are probably MAPs or tubulin molecules connected with MAPs. In addition, we occasionally observed filamentous structures spreading out from microtubule bundles (Fig. 4E). Their diameters were also much smaller than those of microtubules. Another unique structure we observed was a mass of small particles. These masses were observed at the distal tip of a haptonema and usually existed as a pair (Fig. 4F), suggesting a cap structure at the distal end of haptonematal microtubules. Alternatively, they may simply be masses of tubulins depolymerized from the distal part of haptonematal microtubules because they are structurally similar to the depolymerized microtubules (Fig. 4G).

### Taxol inhibits haptonematal coiling

**Fig. 5.**
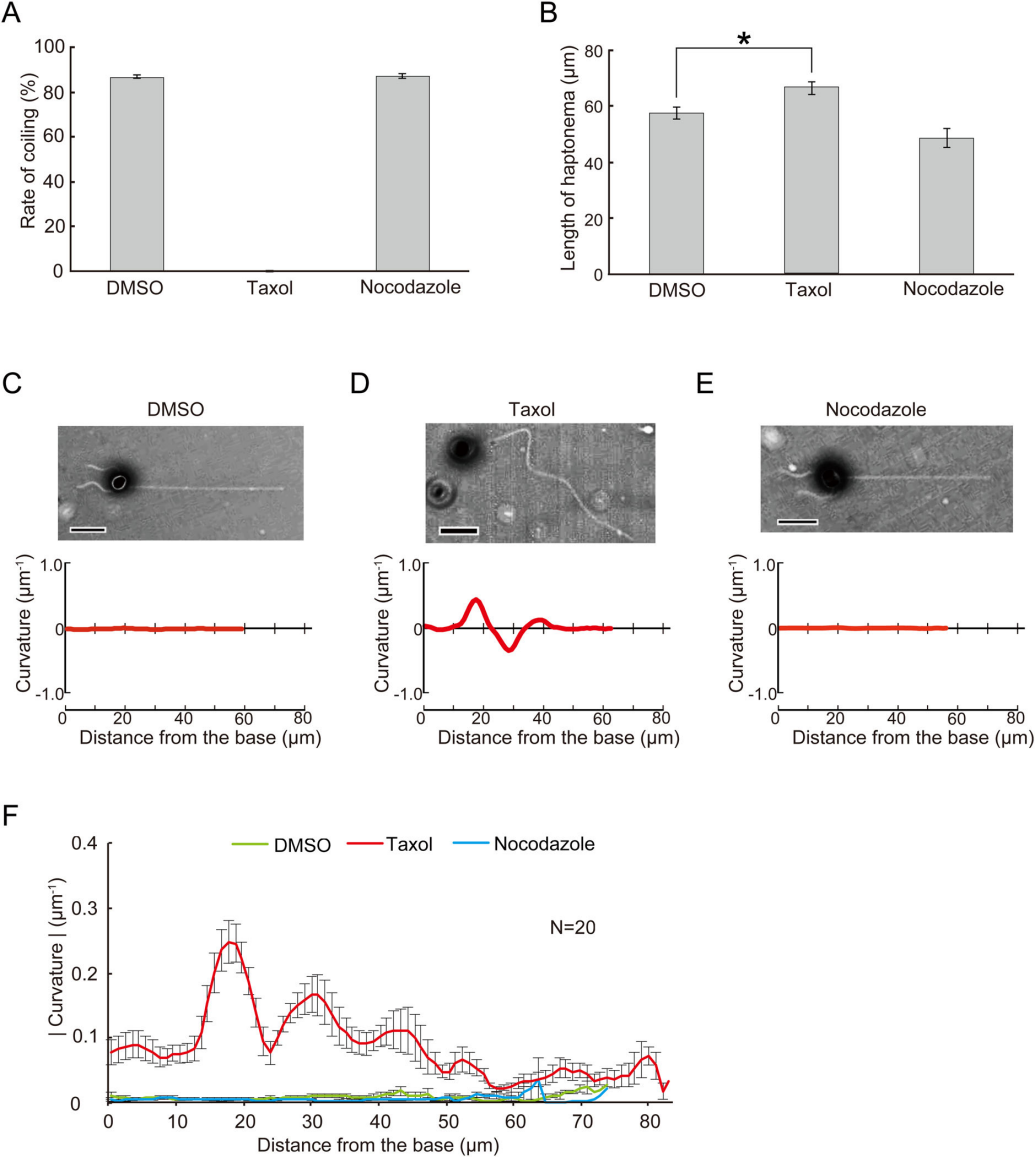
**Effects of drugs that affect microtubules on haptonematal coiling.** Haptophytes were suspended in artificial sea water containing 9.18 mM CaCl_2_ in the presence of drugs. After 60 min, haptonematal coiling was induced by tapping of the microscopic stage. (A) Rates of coiling in the presence of taxol (20 μM) or nocodazole (20 μM). Error bars show the standard deviation. *N*=5. (B) Length of haptonemata at 60 min after treatment with each drug. Error bars show the standard deviation. *N*=5. The asterisk represents that the difference is significant at *P*<0.01 (Student's *t*-test). (C–E) Effects of each drug on the curvature along the axis of a haptonema. The curvature was measured as described in Fig. S5, from the phase-contrast images shown above each plot. Scale bar: 10 µm. (F) Absolute values of the curvature at 60 min after incubation, showing the extent of bending along the haptonema. Error bars show the standard deviation. *N*=20.

Ultrastructural observation of the haptonema indicated dynamic structural changes between coiled and extended states. To examine the possibility that microtubule dynamics are involved in the coiling mechanism, we used taxol and nocodazole, which inhibit microtubule depolymerization and polymerization, respectively. We incubated the haptophytes with each drug for 60 min and induced coiling by tapping the microscope stage. Nearly 80% of nocodazole-treated and control haptophytes showed coiling. Another microtubule drug that inhibits microtubule polymerization, colchicine, also caused no inhibition of coiling (Fig. S3). However, haptophytes treated with taxol showed significantly inhibited coiling (Fig. 5A). In the extended state without mechanical stimulation, control haptonemata remained straight but taxol-treated haptonemata showed planar bending with low curvatures. The overall length of taxol-treated haptonema was significantly increased (Fig. 5B), which might indicate elongation of the microtubules, possibly from the tubulin pool at the distal tip. Decreased length was observed for nocodazole or colchicine-treated haptonemata but this was not significant. To confirm if these microtubule drugs effectively work in the haptophyte *Chrysochromulina* sp. NIES-4122, we checked the growth rate in the presence of these drugs. Cell growth was suppressed by all three microtubule drugs, indicating that the drugs work on mitotic apparatus at the concentrations used in experiments (Fig. S4).

To quantitatively evaluate the taxol-induced bending, we measured the curvature along the haptonema (Fig. S5A). The curvature was almost zero in control, nocodazole-treated and colchicine-treated haptophytes throughout the haptonema (Fig. 5C,E), but taxol-treated haptonema showed a large peak in the proximal region with the maximum at around 15–20 μm from the base (Fig. 5D,F; Fig. S5B). This bending was initially formed in the distal to middle region 5 min after treatment and then propagated toward the proximal region (Fig. S6A).

### Changes of taxol-induced haptonema bending by Ca^2+^

**Fig. 6.**
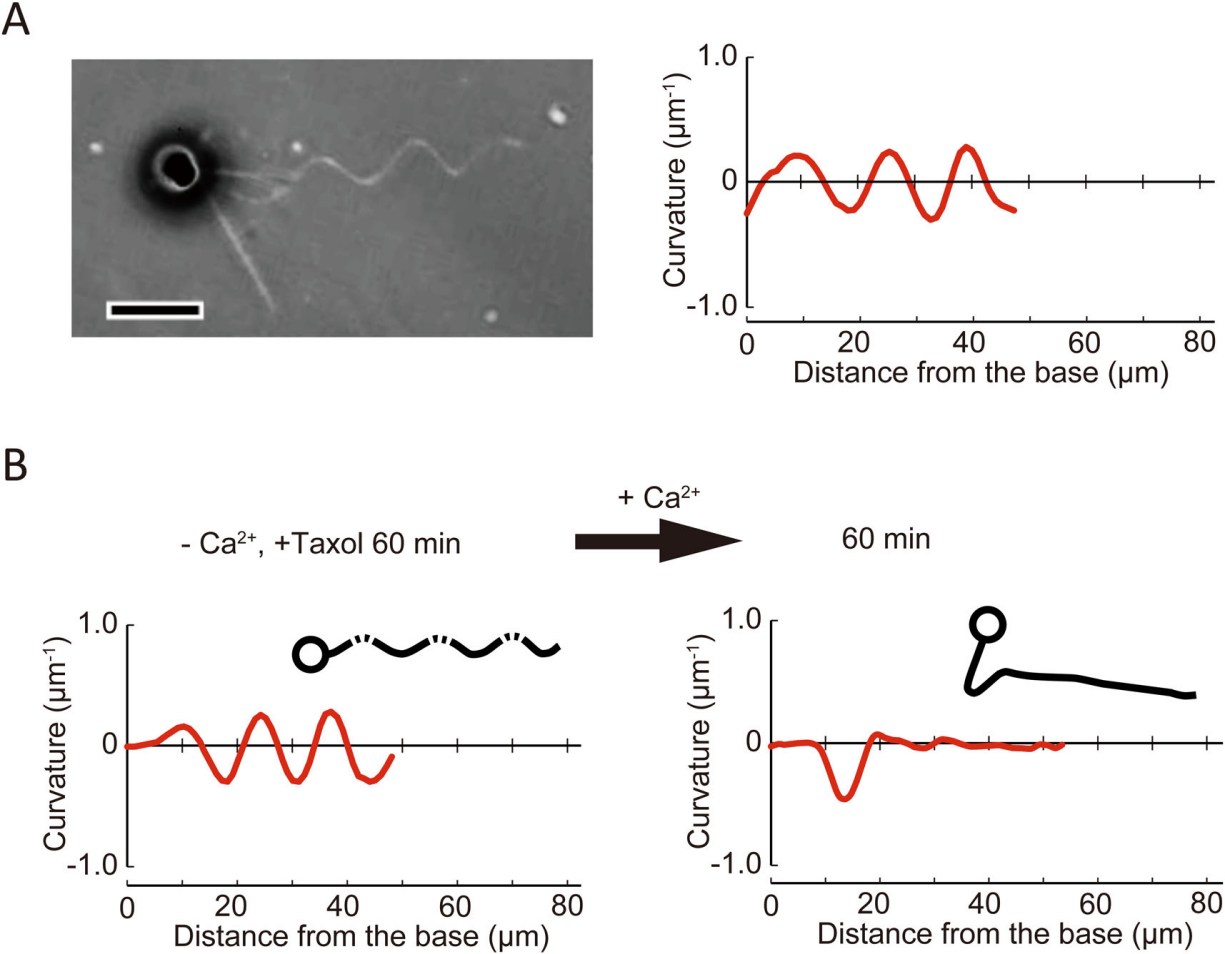
**Effects of Ca^2+^ on taxol-induced haptonematal bending.** (A) A phase contrast image of a haptophyte treated with taxol (20 μM) in the absence of Ca^2+^ (CFSW+EGTA+BAPTA-AM) showing a helical configuration of the haptonema (left). The plot to the right shows the curvature along the haptonema (see Fig. S5). The curvature of the haptonema was calculated from the microscopic image, which is a two-dimensionally projected image. The curvature of a helical structure calculated in this way shows oscillation with a repeat distance equivalent to that of the helix, and a shift in the pattern represents propagation of the wave form. Scale bar: 10 µm. (B) Ca^2+^-dependent propagation of a taxol-induced haptonematal helix. The helix is also converted to planar bending with the maximum curvature of bend propagating to the proximal region. Trace images of haptophytes are shown as insets.

To explore the relationship between the mechanism of coiling and taxol-induced bend formation, we examined the effect of Ca^2+^ on haptonemata after treatment with taxol in Ca^2+^-free conditions. In contrast to taxol-treated haptonemata in normal sea water with Ca^2+^ (Fig. 5D), the haptonema in Ca^2+^-free conditions did not show planar bending but rather twisted or helical shapes (Fig. 6A). Careful observation by altering the microscope focus showed that the helix was right-handed. The helix was maintained from 5 min to 60 min but became gentle in pitch to extend toward the tip (Fig. S6B).

To examine the requirement of Ca^2+^, taxol-treated haptonemata were first incubated in Ca^2+^-free conditions containing EGTA/BAPTA-AM for 60 min, and then 50 mM CaCl_2_ was added. The twist observed 60 min after the incubation in Ca^2+^-free conditions gradually became planar and shifted toward the base of the haptonema after the addition of CaCl_2_ (Fig. 6B). The final waveform became similar to that observed in taxol-containing artificial sea water (Fig. 5D).

## DISCUSSION

Several non-motor, unconventional types of movements are known in protists, such as the contraction of heliozoan axopodia (Tilney and Porter, 1965; MacDonald and Kitching, 1967; Suzaki et al., 1980; Hausmann et al., 1983) and spasmoneme contraction in peritrichous ciliates (Amos et al., 1975). The former is induced by Ca^2+^-dependent cataclysmic breakdown of microtubules as well as their depolymerization (Febvre-Chevalier and Febvre, 1986) and the latter is caused by conformational change of helically coiled structures, mainly constructed of a 20 kDa centrin-related Ca^2+^-binding protein, called spasmin (Amos et al., 1975; Asai et al., 1978; Misra et al., 2010). Haptonematal coiling is a unique type of microtubule-dependent motility, distinct from these known non-motor motile systems in eukaryotes.

From a series of experiments and observations, we obtained two important insights regarding the microtubule configuration in haptonematal coiling. First, we showed that the microtubules in the coiled haptonema have highly curved structures. The values of curvature estimated here are equivalent to or over the threshold for microtubule breakage (Odde et al., 1999). Therefore, it is likely that haptonematal microtubules need extra structural reinforcement by MAPs, as observed in EM (Fig. 4D), to prevent them from breakage during coiling. From the analysis of TEM images, seven microtubules run in parallel in the extended haptonema (Fig. 3E) and mostly in parallel except one microtubule in the coiled haptonema (Fig. 3F,G). Microtubules in the coiled haptonema show not a simple coiling but writhing with microtubules crossed over at two positions in a coil (Fig. 4C). This configurational change is closely analogous to the twist-writhe conversion of erupting filaments or prominences in the solar corona, explained by the line tied, cylindrically symmetric Gold–Hoyle flux rope model (Gold and Hoyle, 1960; Török et al., 2014; Fig. S7).

Second, we found that taxol inhibited the rapid coiling of a haptonema (Figs 5,6). This inhibition is not by the inhibition of microtubule depolymerization (Fig. 4). Taxol binds to β-tubulin, resulting in a longitudinal extension of the αβ-tubulin dimer spacing, formation of straight protofilaments and stabilization of microtubules (Arnal and Wade, 1995; Elie-Caille et al., 2007; Mitra and Sept, 2008). Microtubules stabilized with taxol become more flexible than microtubules without taxol (Felgner et al., 1996; Venier et al., 1994; Kikumoto et al., 2006; Mitra and Sept, 2008), although the opposite result was also reported (Mickey and Howard, 1995). Therefore, the helix of a haptonema formed in the presence of taxol and absence of Ca^2+^ (Fig. 6) is likely to be caused by changes in the mechanical and structural properties of microtubules. Because taxol alters the tubulin lattice and supertwist of microtubules (Arnal and Wade, 1995), each microtubule of the haptonema is considered to be distorted by taxol treatment in the absence of Ca^2+^. Bundling of microtubules by MAPs would coordinate and convert the distortion into the right-handed gentle helix of the haptonema (Fig. 6A). Addition of Ca^2+^ to a taxol-treated haptonema disrupts this helix and causes formation of a planar bend (Fig. 6B), implying that MAPs respond to Ca^2+^ and change the helical configuration (Fig. S7). Nevertheless, a taxol-treated haptonema never shows left-handed coiling, probably because another configurational change of haptonematal microtubules is essential to cause coiling via Ca^2+^-dependent conformational changes of MAPs without taxol.

Taken together, we consider a model for haptonematal coiling, based on the twist-writhe conversion by the aid of Ca^2+^-binding MAPs (Fig. S7). In this model, structural changes of MAPs caused by Ca^2+^ binding induce a mechanical strain on the microtubules (Fig. S7B; high energy state), which is relieved by coiling of the haptonema (Fig. S7C; low energy state). When Ca^2+^ is released, the resulting structural change of the MAPs would make the coiled structure unstable (Fig. S7D), and the haptonema resumes the extended form (Fig. S7A). Some MAPs are known to cause conformational changes in protofilaments and overall microtubule structures, as seen in the case of stathmin (Sobel et al., 1989; Brouhard and Rice, 2014). The combination of taxol and MAPs has novel effects on the mechanical properties of microtubules (Hawkins et al., 2013), which is consistent with our present data. Further studies on the structures and Ca^2+^-dependent configurational changes of haptonematal microtubules and their associated proteins using cryoelectron microscopy and tomography should shed light on the mechanism of rapid coiling induced without microtubule-based motors.

## MATERIALS AND METHODS

### Haptophytes

*Chrysochromulina* sp. was isolated from Tokyo bay in 2013 using micropipette isolation (Anderson and Kawachi, 2005). It was then characterized (Fig. S1) and registered as a culture collection (NIES-4122). *Chrysochromulina* sp. NIES-4122 was maintained in Daigo IMK medium (Nippon Seiyaku Co., Osaka, Japan) at 20°C under a 14/10 h light/dark regime. The haptophyte could be recovered as a pellet after centrifugation but the haptonemata were mostly detached owing to the mechanical stimulus of colliding with the bottom of the centrifuge tube. To increase the number of cells in a microscopic field, we concentrated haptophytes using a non-toxic density gradient medium, Percoll. Percoll (GE healthcare) was diluted with two times concentrated artificial seawater (ASW; 460.3 mM NaCl, 10.11 mM KCl, 9.18 mM CaCl_2_, 35.91 mM MgCl_2_, 17.49 mM MgSO_4_, 0.1 mM EDTA and 10 mM HEPES-NaOH, pH 8.2) to make 50% Percoll. 10 ml of culture was layered on 100 μl of 50% Percoll in a 15 ml Falcon tube and was centrifuged at 2800 ***g*** for 2 min. 100 μl of concentrated cells were collected from the boundary between the culture medium and Percoll.

### Light microscopy observation of haptonematal coiling and uncoiling

Coiling and uncoiling were induced by the tapping of the microscopic stage (Kawachi and Inouye, 1994). Haptonemata were observed with a phase contrast microscope (BX51, Olympus, Tokyo, Japan). To record haptonematal coiling, the microscope was connected to a high-speed CCD HAS-D3 camera (Ditect, Tokyo, Japan). Haptonematal bending was analyzed from high-speed camera images using Bohboh software (Bohboh Soft, Tokyo, Japan). To examine the effects of Ca^2+^, 100 µl of concentrated cells were mixed with 900 µl of Ca^2+^-free ASW (462.01 mM NaCl, 9.39 mM KCl, 59.08 mM MgCl_2_, 10 mM HEPES, pH 8.0), 10 mM EGTA in Ca^2+^-free ASW, or 10 µM EGTA and 50 µM BAPTA-AM (Dojindo, 50 mM stock solution in DMSO) in Ca^2+^-free ASW. Paclitaxel (taxol) and nocodazole were dissolved in dimethyl sulfoxide (DMSO) to 20 mM and added to media to a final concentration of 20 µM.

### Electron microscopy

Thin-section electron microscopy was performed as described previously (Konno et al., 2010) with some modifications. To examine microtubule configurations in sequential images, a flat surface of polymerized Epon resin was treated with 1 mg/ml poly-lysine for 30 min, followed by coating with 10 mg/ml bovine serum albumin (BSA). Harvested haptophytes were fixed with 2.5% glutaraldehyde and were then deposited onto the poly-lysine coated surface by centrifugation at 200× ***g*** for 10 min. The samples were post-fixed with 1% OsO_4_, dehydrated and embedded again according to a general procedure for thin-section electron microscopy.

For negative staining, we first tried to remove the plasma membrane by NP-40 in a tube before depositing the haptonema to a grid. However, we could not see enough numbers of haptonemata on an EM grid, possibly due to structural disruption of the microtubule structure during the treatment. Demembranation on the grid after attachment of intact haptonema helped structural maintenance. Harvested cells were incubated in Ca^2+^-free ASW containing 10 mM EGTA for 10 min. Cells were then gently mounted on a carbon-coated Cu grid and demembranated with 0.5% NP-40 in 100 mM HEPES-NaOH (pH 7.2), 2 mM MgSO_4_, 5 mM EGTA for 15 s. The grid was rinsed with the HEPES buffer for 1 min and negatively stained with 1% Uranyl Acetate. Electron microscopy was carried out using a JEOL 1010 or Tecnai F20 transmission electron microscope.

### Estimation of haptonematal curvature

The curvature of coiled haptonemata was calculated from negatively stained EM images and light microscopic images obtained using a differential interference contrast system with 100× objective (Olympus UPlanApo 100×/1.35 NA), according to the three-point method described in Odde et al. (1999) and Bicek et al. (2007). The coordinates of the points along the center line of the haptonema were recorded at the intervals of 26–115 nm for EM images and 0.24 µm for the light-microscopic images, respectively. Alternatively, the curvature at each point along the full length of an extended haptonema was simply calculated as the reciprocal radius of the inscribed circle as described previously (Mizuno et al., 2012). Note that these curvature values were calculated from the EM or light-microscopic images, which are two-dimensional projection of the three-dimensional structures.

## Supplementary Material

Supplementary information
