# Supplementary material for: Microtubule stabilizer reveals requirement of Ca2+-dependent conformational changes of microtubules for rapid coiling of haptonema in haptophyte algae
Source: Biol Open. 2019 Jan 30;8(2):bio036590. doi: 10.1242/bio.036590 (PMC6398456; doi:10.1242/bio.036590)
Supplement: Supplementary information [file biolopen-8-036590-s1.pdf]

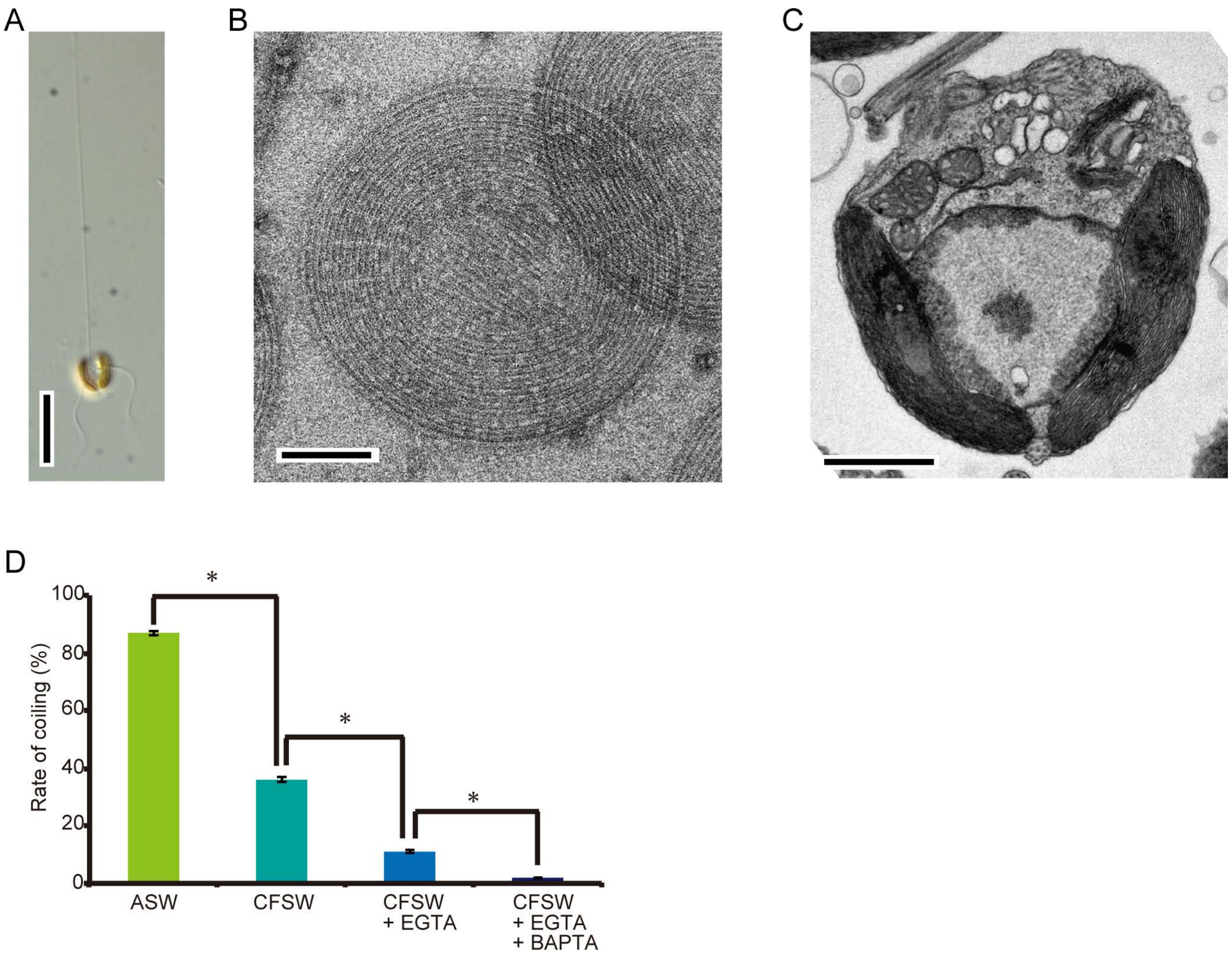

Fig. S1. Characterization of morphology and haptonematal coiling in *Chrysochromulina* sp. NIES-4122.

(**A**) Negative staining image showing extracellular scales without spines. Bar, 100 nm. (**B**) A differential interference contrast image of a whole haptophyte. Bar, 10 µm. (**C**) Thin section electron microscopy image of the cell body. Bar, 1 µm. (**D**) Ca^{2+}-dependent haptonematal coiling. Haptophytes were suspended in artificial sea water (ASW), Ca^{2+}-free sea water (CFSW), CFSW with 10 mM EGTA, or CFSW with 10 mM EGTA plus 50 µM BAPTA-AM. Error bars represent the standard deviation. N=5. *P<0.01 (Student's test).

Biology Open • Supplementary information

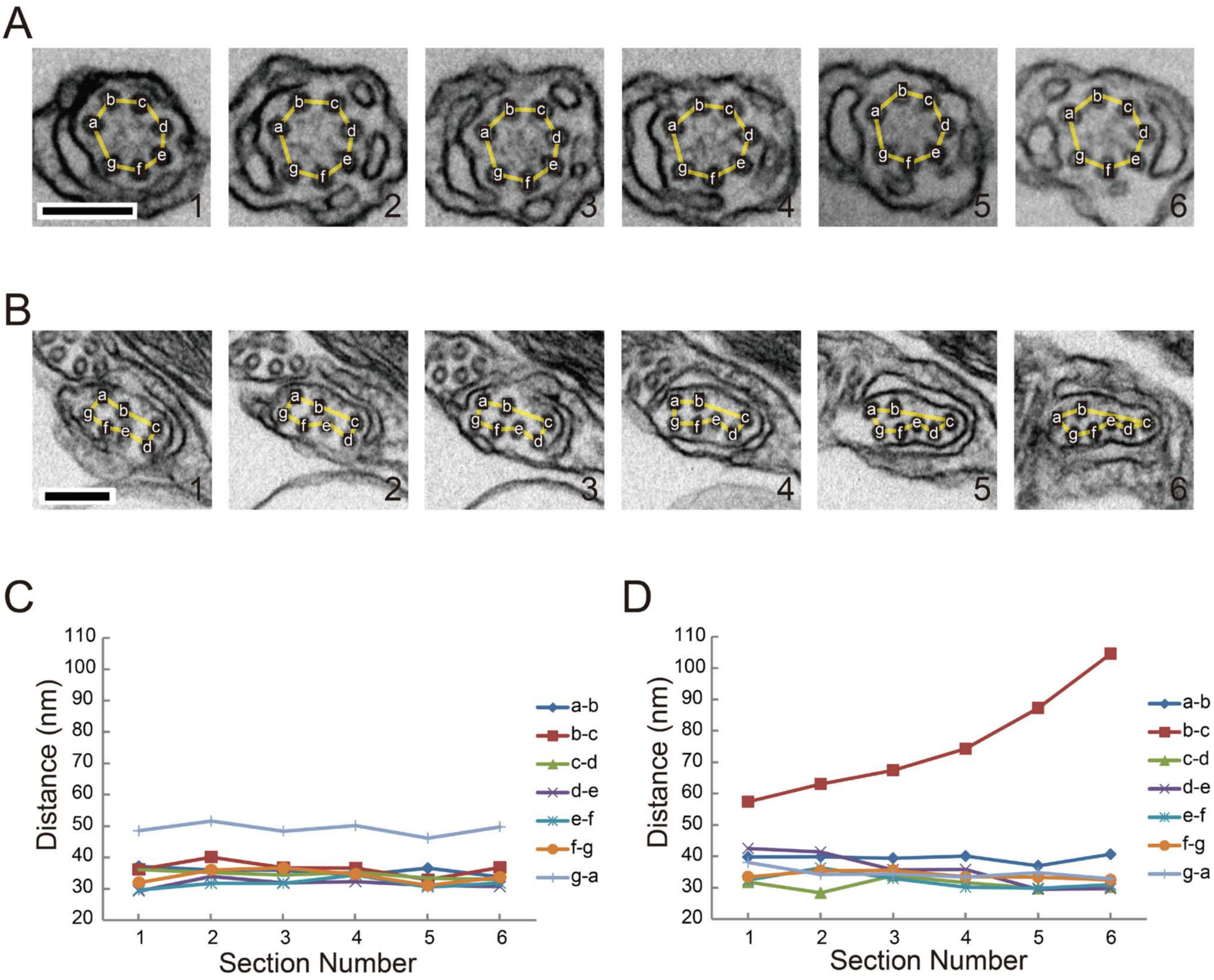

Fig. S2. Changes in the center-to-center distance between adjacent haptonematal microtubules.

The center-to-center distances between adjacent microtubules were measured from six sequential thin-section electron microscopy images. (**A, B**) Sequential thin-section images of an extended (A) and coiled (B) haptonemata. The section numbers and the letters on the microtubules correspond to those in C and D. Bars, 100 nm. (**C, D**) Center-to-center distances between adjacent microtubules were plotted against the sequential section number, showing changes in the distance along the longitudinal axis of the haptonema. The extended haptonema (**C**) shows relatively constant distances among sequential sections but the coiled haptonema sometimes shows deviation for one of the inter-microtubule distances, which changes with the section number (**D**).

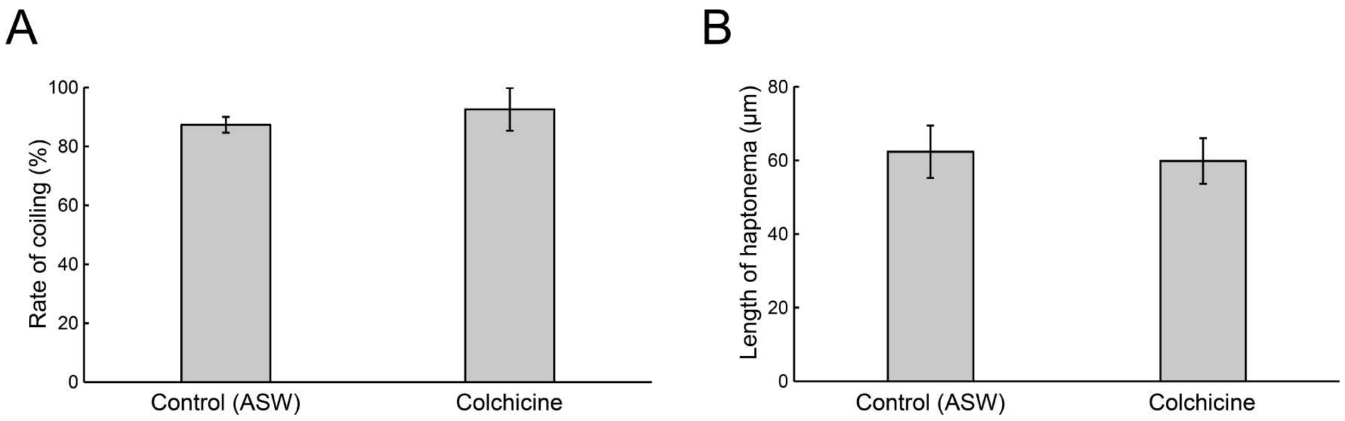

Fig. S3. Effects of colchicine on haptonematal coiling.

(A) Rates of coiling in the presence of 1 mM colchicine. Error bars show the standard deviation. N = 5. (**B**) Length of haptonemata at 60 min after colchicine treatment. Error bars show the standard deviation. N = 5.

Biology Open • Supplementary information

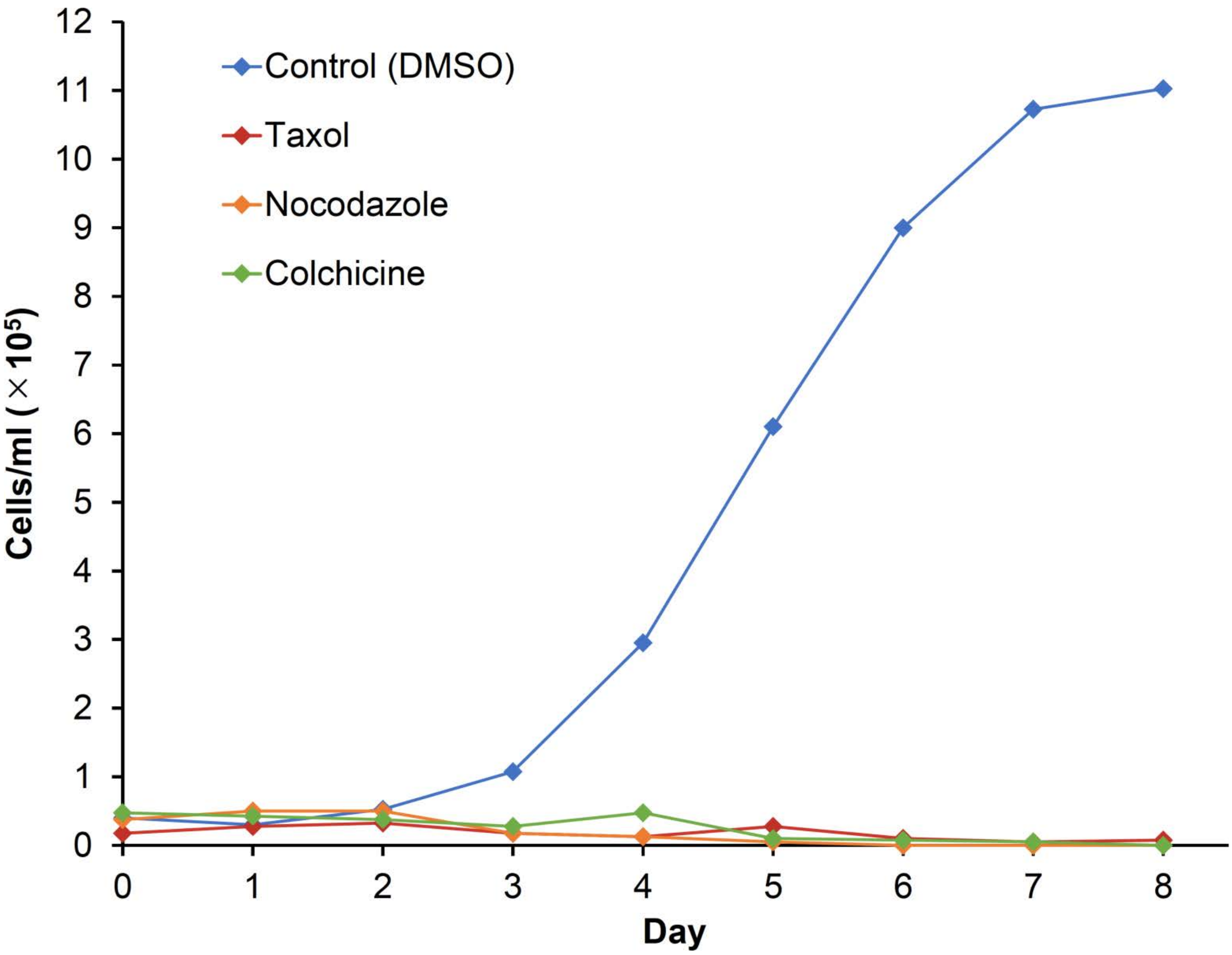

Fig. S4. Effects of microtubule drugs on the growth of *Chrysochromulina* sp. NIES-4122.

Cells (1.8–4.8 x 10^4 cells/ml) were cultured in Daigo IMK medium in the presence of 20 μM taxol, 20 μM nocodazole or 1 mM colchicine. The culture without drugs (only solvent; DMSO) was used as the control. The number of cells was counted every day for 8 days at 20°C under a 14/10 h light/dark regime. All three drugs significantly suppressed the cell growth.

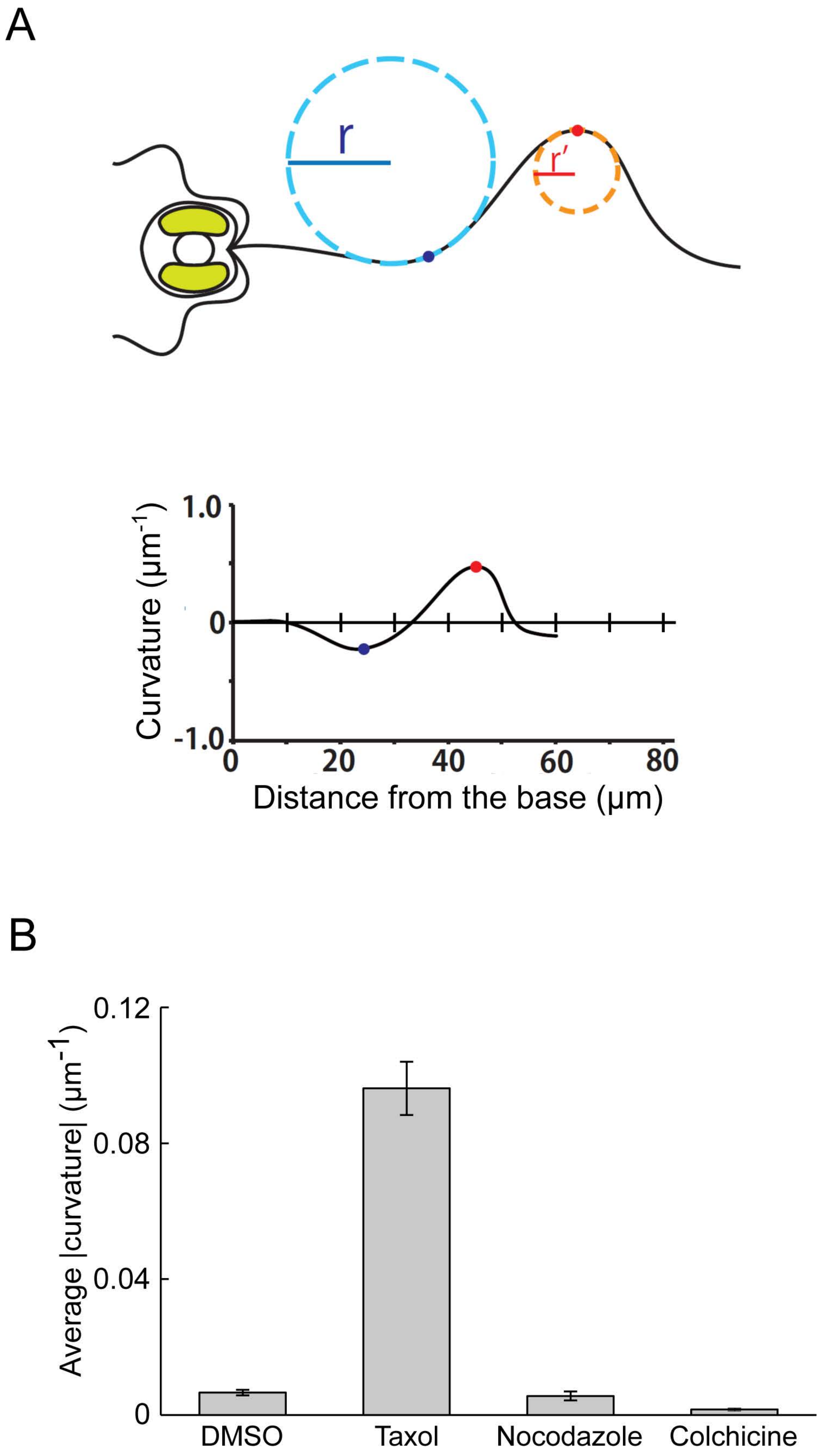

Biology Open • Supplementary information

Fig. S5. Curvature analysis of the haptonema.

(**A**) Top, the curvature of a certain region of a haptonema was defined as the reciprocal of the radius of the inscribed circle (dashed blue or orange line). Bottom, an example of the curvature plot against distance from the base of a haptonema. The curvature values are either positive or negative, depending on the bending direction. The absolute value of curvature is used in Fig. 5F and Fig.S5 to show the extent of bending. (**B**) Comparison of the maximum curvature of bends induced by microtubule-modifying drugs. Based on the analysis in Fig. 5 and Fig. S3, the average of absolute curvature values was calculated. Bars represent the standard deviation. N = 20.

Biology Open • Supplementary information

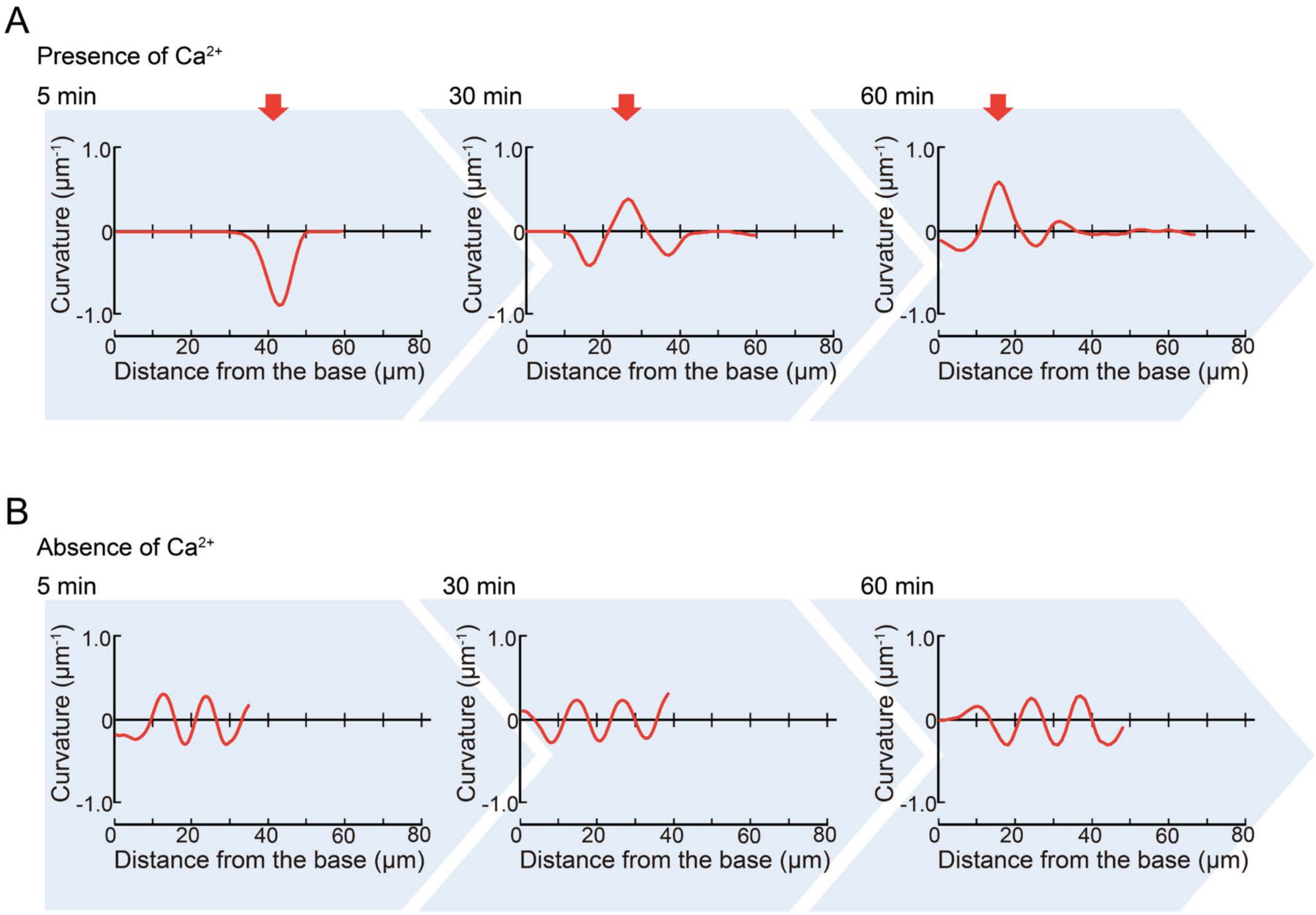

Fig. S6. Bend propagation of taxol-treated haptonemata.

The curvature along the haptonema was measured at 5, 30 and 60 min after treatment with 20 μM taxol. (**A**) In the presence of Ca^{2+} (ASW); (**B**) In the absence of Ca^{2+} (CFSW+EGTA+BAPTA-AM). Note that the peak of the maximum curvature (arrows) propagates toward the base of the haptonema with time in the presence of Ca^{2+} and that the gentle helix propagates toward the tip of the haptonema with time in the absence of Ca^{2+}.

Biology Open • Supplementary information

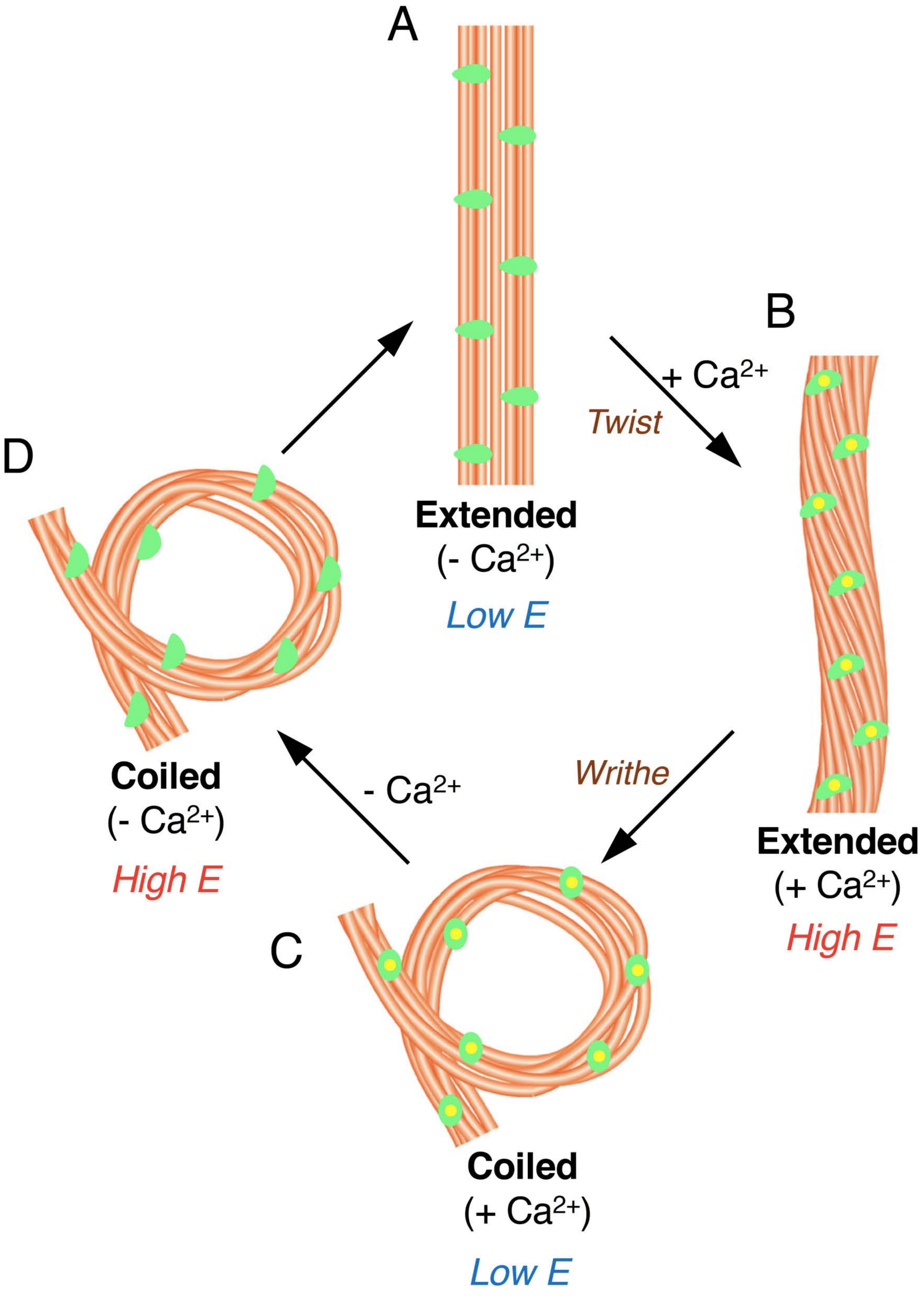

Biology Open • Supplementary information

Fig. S7. A model for configuration changes in haptonematal microtubules during coiling and uncoiling process.

(A) When Ca^{2+} is not bound to the MAPs (green) in the extended state of haptonema, the microtubules are stable in the straight conformation. (B) By binding Ca^{2+} (yellow), the MAPs change their structures, causing twist of the microtubules with instable tension (high energy state). (C) The tension is released (low energy state) when the microtubule bundle writhes to form a coil (see Fig. 4C). (D) When Ca^{2+} is released from the MAPs, their structure changes to a conformation different from the Ca^{2+}-bound form in B. This conformational change brings the microtubule bundle instable again (high energy state) and induces uncoiling of the haptonema. This model is based on the line tied, cylindrically symmetric Gold–Hoyle flux rope model (Gold and Hoyle, 1960; Török et al., 2014), which gives account for the twist-writhe conversion.

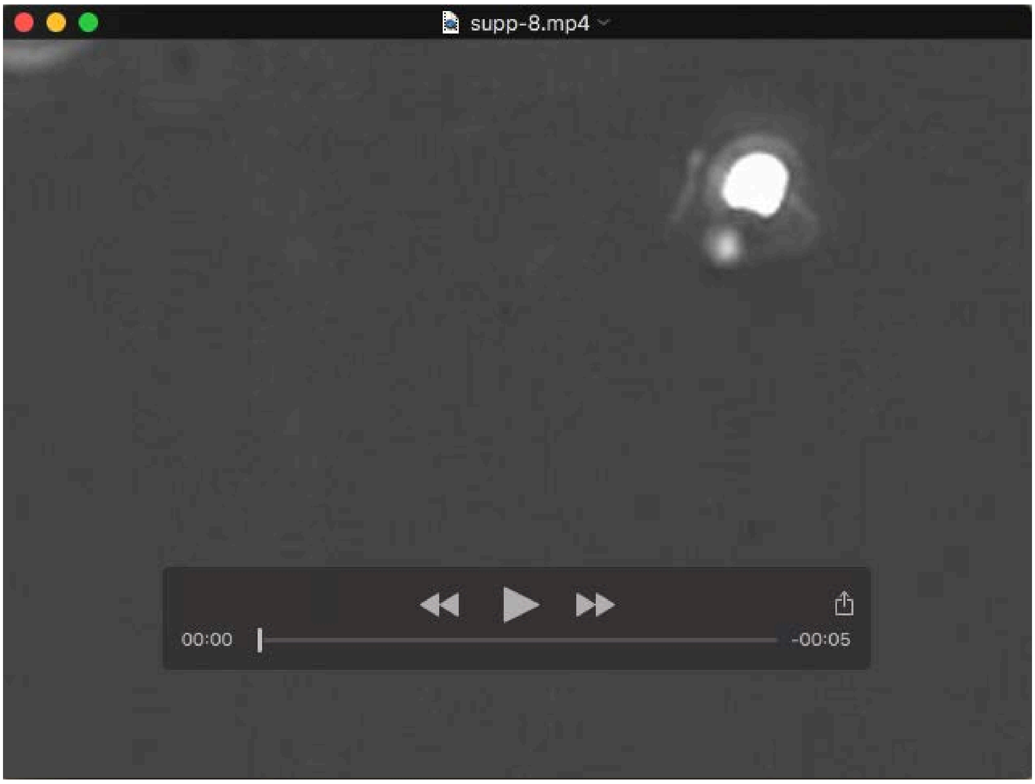

Movie 1. Real-time movie of haptonematal coiling and uncoiling.

The coiling and uncoiling were induced by tapping the microscopic stage. The movie was recorded at 100 fps and plays at real time. The process of coiling is too rapid to be seen but that of uncoiling proceeds more slowly.

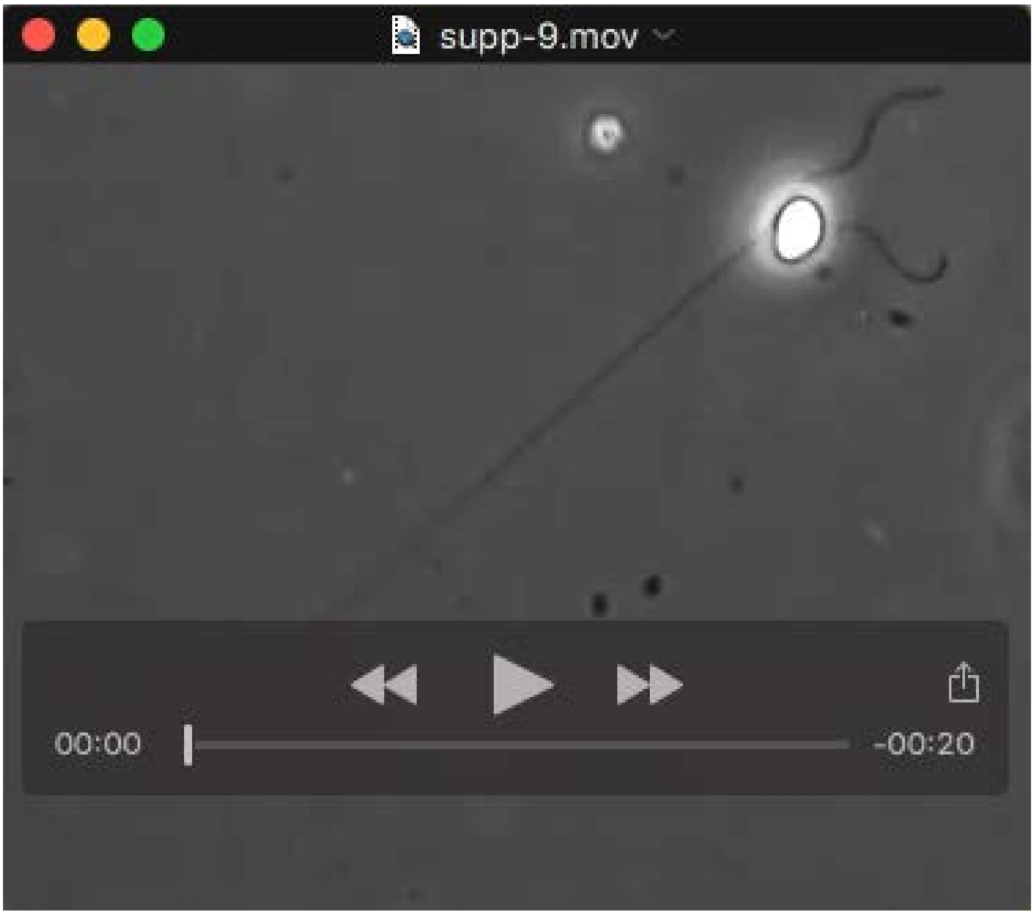

Movie 2. High-speed movie of haptonematal coiling.

The movie was recorded at 1000 fps and plays at a speed of 0.03×.

Biology Open • Supplementary information

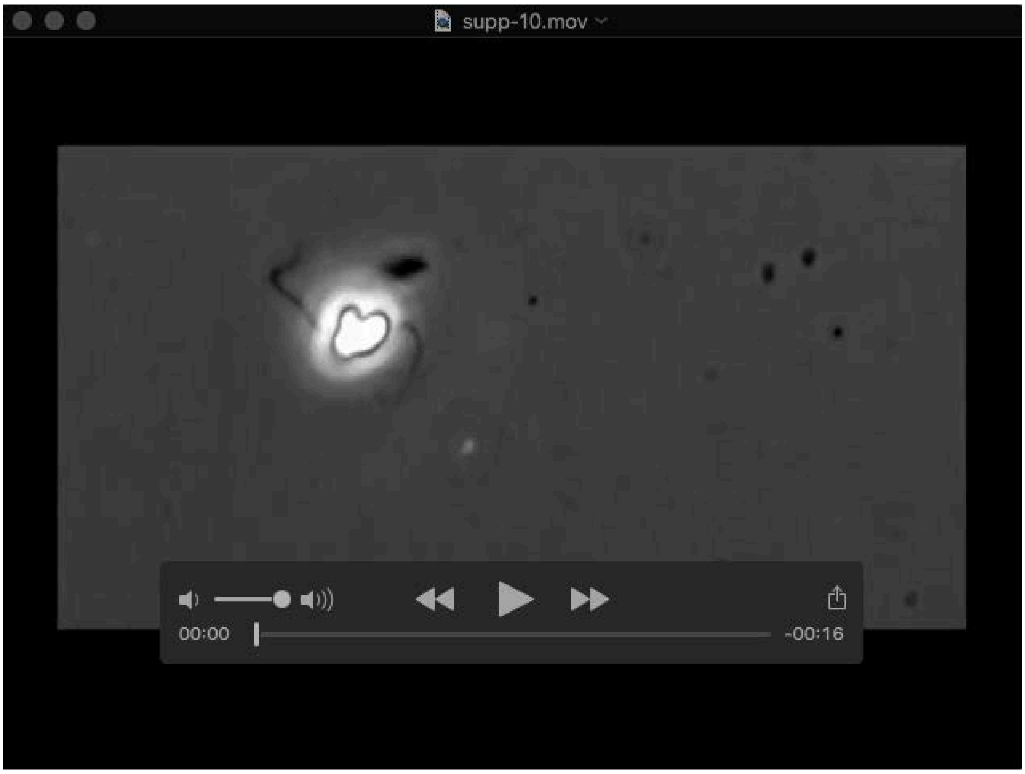

Movie 3. High-speed movie of haptonematal uncoiling.

The movie was recorded at 200 fps and plays at a speed of 0.15×.
